# From Early Immunomodulatory Triggers to Immunosuppressive Outcome: Therapeutic Implications of the Complex Interplay Between the Wavebands of Sunlight and the Skin

**DOI:** 10.3389/fmed.2018.00232

**Published:** 2018-09-10

**Authors:** Pablo A. Vieyra-Garcia, Peter Wolf

**Affiliations:** Department of Dermatology, Medical University of Graz, Graz, Austria

**Keywords:** immunosupression, phototherapy, DNA damage, apoptosis, psoriasis, CTCL

## Abstract

Phototherapy is an efficient treatment for many cutaneous diseases that involve the activation of inflammatory pathways or the overgrowth of cells with aberrant phenotype. In this review, we discuss recent advances in photoimmunology, focusing on the effects of UV-based therapies currently used in dermatology. We describe the molecular responses to the main forms of photo(chemo)therapy such as UVB, UVA-1, and PUVA that include the triggering of apoptotic or immunosuppressive pathways and help to clear diseased skin. The early molecular response to UV involves DNA photoproducts, the isomerization of urocanic acid, the secretion of biophospholipids such as platelet activating factor (PAF), the activation of aryl hydrocarbon receptor and inflammasome, and vitamin D synthesis. The simultaneous and complex interaction of these events regulates the activity of the immune system both locally and systemically, resulting in apoptosis of neoplastic and/or benign cells, reduction of cellular infiltrate, and regulation of cytokines and chemokines. Regulatory T-cells and Langerhans cells, among other skin-resident cellular populations, are deeply affected by UV exposure and are therefore important players in the mechanisms of immunomodulation and the therapeutic value of UV in all its forms. We weigh the contribution of these cells to the therapeutic application of UV and how they may participate in transferring the direct impact of UV on the skin into local and systemic immunomodulation. Moreover, we review the therapeutic mechanisms revealed by clinical and laboratory animal investigations in the most common cutaneous diseases treated with phototherapy such as psoriasis, atopic dermatitis, vitiligo, and cutaneous T-cell lymphoma. Better understanding of phototherapeutic mechanisms in these diseases will help advance treatment in general and make future therapeutic strategies more precise, targeted, personalized, safe, and efficient.

Sunlight and its wavebands profoundly affect the cellular physiology and dynamics of the skin. Exposure to ultraviolet radiation (UVR) leads in the short term to sunburn and tanning and in the long term to photoaging and carcinogenesis. However, it is also well known that UVR exposure can benefit patients with certain skin diseases including psoriasis, atopic dermatitis, and cutaneous T-cell lymphoma (CTCL). The initial triggers for these diverse effects of UVR include DNA damage (1); cis-to-trans urocanic acid (UCA) isomerization (2); formation of active biophospholipids such as platelet activating factor (PAF) (3); and activation of aryl hydrocarbon receptor (AhR), inflammasome, and/or oxidative stress-related enzymes such as nitric oxygen synthase (NOS) (4, 5), Subsequent activation of apoptosis and mechanisms of local and systemic immunosuppression helps to counteract the effects of UV. After UV exposure, keratinocytes, melanocytes, and immune cells that reside in the skin, increase the release of cytokines such as TNF-α, IL-6, and IL-10 (6); chemokines such as CCL27 and IL-8 (7); and metabolic products such as vitamin D, that are involved in the onset of local and systemic effects of UV in complex regulatory loops. Langerhans cells (LC) and other dendritic cells as well as regulatory T-cells (Tregs) migrate in and out of the skin, thereby coordinating a series of crucial events for the establishment of an immunosuppressive microenvironment (8).

Last year, the many efforts to define the role of visible light in the complex interplay between UVR and living organisms received recognition when JC Hall, M Rosbash, and MW Young were awarded the Nobel Prize in medicine and physiology for their work on the genes that control circadian rhythm. Their work showed that proteins such as PER or TIM in fruit flies (9) and later CLOCK in mammals (10) accumulate during the night and degrade during the day in a self-regulatory feedback loop that establishes a neuronally regulated central clock-like system. In daytime, the skin is constantly exposed to UVR. The circadian rhythm pathways affect the skin's handling of UVR effects through cooperative or autonomous processes such as vitamin D synthesis, reactive oxygen species (ROS) production, DNA damage, cell senescence, and immunosuppression. One example of this influence on cutaneous dynamics is seen in mice whose food intake is restricted to certain times during the circadian cycle: alterations in biological clock genes like PER2 lead to a shift of up to 10% in the cutaneous transcriptome of animals under this food intake regime (11). Additionally, genes that mitigate photo-induced DNA damage like XPA are less active during the day in mice with high nocturnal food intake, resulting in prominent accumulation of cyclobutane pyrimidine dimers (CPD) induced by diurnal experimental UVR (11). Wound healing is also controlled by the circadian cycle; skin injuries suffered during the day heal faster than those suffered during the night due to a circadian control of actin polymerization regulated by CRY and PER2 proteins (12). Keratinocytes downregulate TIMP3, a metalloproteinase inhibitor linked to CLOCK upon UVR exposure, which in turn leads to an upregulation of MMP1, TNF-α, CXCL1, and IL-8 promoted by C/EBP (a CCAAT-enhancer binding protein) (13). This indicates that UVR affects tissue remodeling and inflammatory signaling pathways by modifying the transcriptional profile of keratinocytes. A recent study looking at the role of circadian proteins in psoriasis found that loss-of-function mutations in CLOCK lead to a less severe psoriatic phenotype in imiquimod-treated mice, whereas PER2 mutations lead to increased expression of IL-23R in γ/δ T-cells in the skin and a more severe psoriatic manifestation (14). If circadian proteins do indeed influence the severity of cutaneous diseases, then the effectiveness of phototherapy may also depend in part on circadian cycles. However, this has not yet been explored.

The physiologic reaction of the skin to UV exposure has been harnessed therapeutically. From the first attempts of Nobel laureate Niels Finsen to treat bacterial infections with UV (15) to the clinical approaches of today in which patients are exposed to UV radiation alone or in combination with photosensitizing agents (i.e., psoralens), (16) phototherapy has provided effective management of cutaneous diseases.

## Sensing of UV exposure and triggering of immunosuppression

### DNA damage

**Figure 1:**
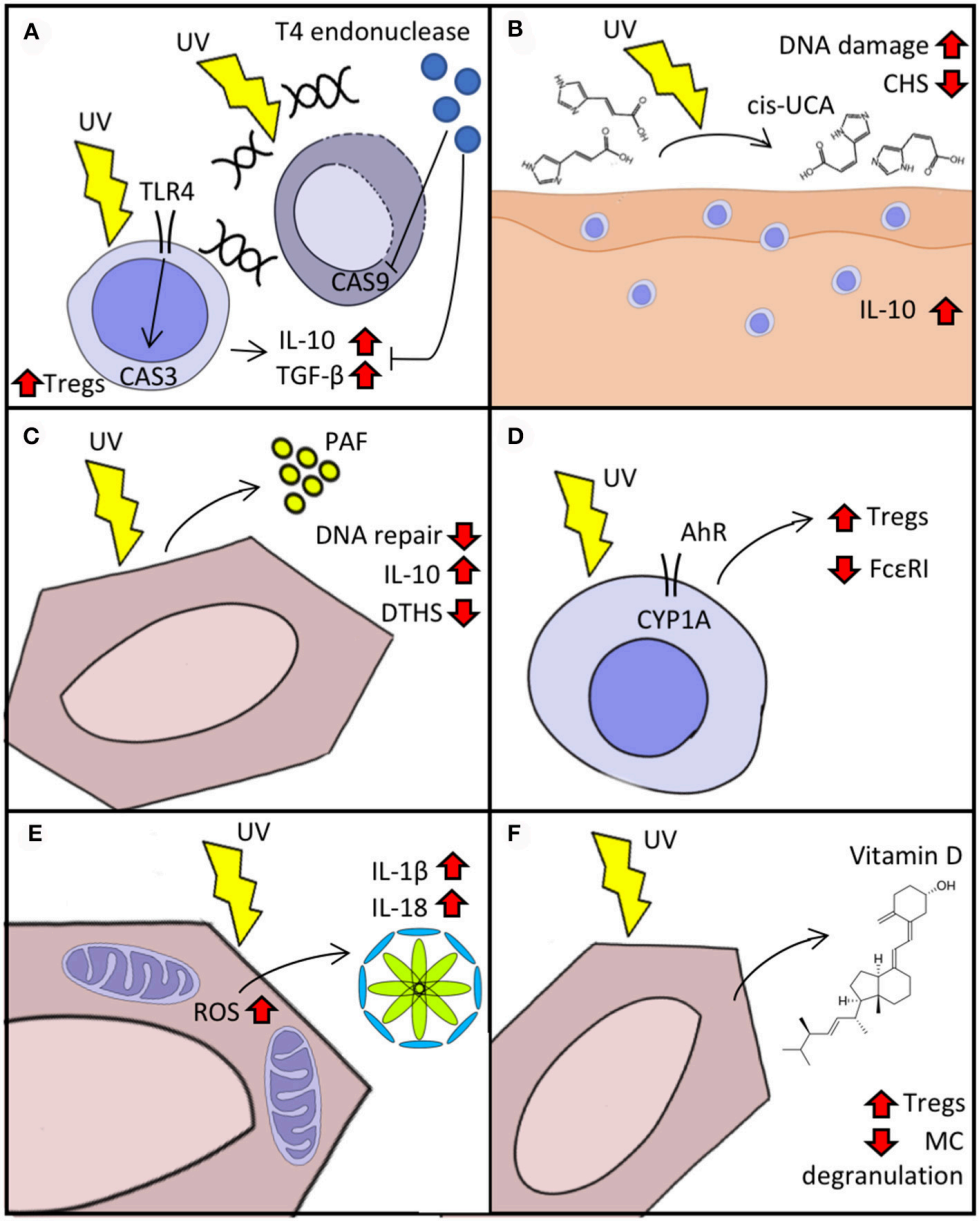
Cellular response to UVR. **(A)** Immunosuppression in response to UV-induced DNA damage mediated by TLR4/MyD88. Delivery of T4 endonuclease decreases caspase (CAS) activation and the “production” of IL-10 and TGF-β. **(B)** Isomerization of urocanic acid (UCA) (trans to cis) after UV exposure increases IL-10 secretion and DNA damage and reduces contact hypersensitivity (CHS). **(C)** Keratinocyte secretion of platelet activating factor (PAF) augments immunosuppression and reduces DNA repair response to UV. **(D)** Activation of aryl hydrocarbon receptor (AhR) after UV reduces expression of FcεRI and boosts Treg activation. **(E)** Reactive oxygen species (ROS) production triggered by UV exposure activates inflammasome in keratinocytes. **(F)** Synthesis of vitamin D after UV exposure activates Tregs and decreases IgE-mediated “mast cell (MC)” degranulation.

Insufficient DNA repair after UVR exposure leads to the accumulation of CPD, which in turn induces immunosuppression and can give rise to skin-tumorigenic gene mutations. The activation of DNA repair mechanisms is modulated in a TLR4/MyD88-dependent manner by the cleavage of the damage-recognition molecule PARP (17). The TLR4/MyD88 axis helps commit UV-exposed cells to apoptosis by activating caspase 3 (18). Experiments with TLR4^−/−^ mice have shown that, after UV exposure, contact hypersensitivity (CHS) responses remain intact in these animals compared to wild type mice and the lymph nodes of TLR4^−/−^ mice have fewer Tregs and lower production of IL-10 and TGF-β (19). This implicates TLR4 not only in the induction of apoptosis but also in the elicitation of immunosuppression after UV exposure. We have shown that the delivery of T4 endonuclease in liposomes to UV-irradiated skin leads to decreased secretion of IL-10 and TNF-α, suggesting that an increased DNA repair capacity can also increase resistance to UV-induced immunosuppression (Figure 1A) (6). Supplementation of IL-12 activates components of the nucleotide-excision repair complex that lower UV-induced DNA damage and prevent immunosuppression (20, 21). The reduced capacity for DNA repair after UV exposure in transplant patients or *in vitro* with immunosuppressive drugs indicate a two-way mechanism (22). Resident memory T-cells (T_RM_) may be implicated in dealing with the effects of UVB. The main function of these cells is to provide surveillance and protection. They participate in wound healing by producing IGF-1 and immunity against pathogens like *Leishmania major* by producing IFN-γ (23, 24). After UV exposure, T_RM_ detect ATP release and increase the production of IL-17, leading to activation of TWEAK (an apoptosis inducer) and GADD45 (a damage-associated cell cycle arrest checkpoint protein), which in turn promote DNA repair (25, 26). Together, these findings highlight the tight interconnection between apoptosis and immunosuppression by means of innate and adaptive immunity and provide a rationale for UV-ameliorating therapies such as DNA repair enzyme supplementation.

### Urocanic acid trans-isomerization

Urocanic acid is synthesized as trans-UCA from histidine in a reaction catalyzed by histidase. It accumulates in the skin at a high concentration (6 nmol/cm in humans) and after UV absorption is isomerized into cis-UCA and contributes to UV-induced immunosuppression (Figure 1B) (2). The treatment of keratinocytes with cis-UCA leads to upregulation of several genes that resemble the transcription profile induced by exposure to UVR, whereas treatment with trans-UCA does not lead to a shift of gene expression (2). Cytokines and proteins that participate in apoptosis, cell cycle arrest, and oxidative stress are upregulated after cis-UCA treatment. Notably, cis-UCA treatment in primary keratinocytes leads to activation of NF-κB and lipid peroxidation, suggesting a complex network of immunomodulatory-related gene transcription (27). The binding of cis-UCA and PAF to their respective receptors (5-HT2A and PAF receptor) contribute to sunburn cell formation, immune suppression, and skin cancer induction upon UVR exposure (28). Moreover, blockade of both cis-UCA and PAF but not vitamin D reduces UV-induced DNA damage in keratinocytes of mouse skin (29).

### Platelet activating factor

Exposure to UVR elicits the secretion of PAF by keratinocytes, which in turn promotes the migration of mast cells into draining lymph nodes where they play an important role in immunosuppression (Figure 1C) (3). After PAF stimulation, mast cells undergo epigenetic modifications that increase their responsiveness to CXCR4 agonists. These modifications are mediated by increased expression of DNMT1/3b (members of a DNA methyltransferase protein family) and p300 (a histone acetyltransferase) and decreased expression of HDAC2 (30). PAF also disrupts DNA-repair mechanisms upon UVR exposure by decreasing the expression of response elements such as MCPH1/BRIT-1 and ATR (31). We have reported that blockade of PAF receptor in mice treated with psoralen plus UVA (PUVA) leads to reduced IL-10 production, less delayed-type immune suppression in response to *Candida albicans*, and lower rates of keratinocyte apoptosis (32).

### Aryl hydrocarbon receptor

CYP1A1 upregulation after UVR exposure implicates AhR in the skin's response to UV (4). AhR-knockout mice lack UVR-induced immunosuppression on CHS challenge (Figure 1D) (33). It has also been shown that AhR participates in the induction of Tregs during T-cell differentiation in the thymus and that certain AhR agonists such as TCDD activate Tregs in the skin and gut (34–36). Moreover, AhR not only participates in Treg-mediated UVR immunosuppression but also decreases the expression of the high affinity receptor for IgE (FcεRI) in LCs and upregulates immunosuppressive molecules such as IDO-1 (37). Atopic dermatitis in human patients is known to flare upon activation of FcεRI in LCs (38), suggesting that therapy with UVB may act by AhR-mediated downregulation of FcεRI.

### Reactive oxygen species and inflammasome

UV-irradiated skin shows immediate changes in a wide range of cellular processes. The biochemistry of keratinocytes and fibroblasts is rapidly redirected to produce ROS by increasing catalase activity and upregulating NOS (5). ROS activate various signaling pathways that involve stress-response factors, for example, the translocation of AP-1 and NF-κB, both of which are under the control of MAPKs that culminate in tissue remodeling and accelerated senescence (39).

ROS generation also activates inflammasome, a multiprotein intracellular oligomer responsible for initiating inflammatory responses by converting IL-1β and IL-18 into their active form and triggering inflammation-dependent cell death (pyroptosis) (Figure 1E) (40). UVB activates NLRP3 inflammasome in keratinocyte cells after sensing UVB-induced DNA damage (1). Yet, despite such inflammasome activation, the effects of UVR on the immune system are predominantly immunosuppressive. Upon UVB exposure, LCs emigrate from the epidermis in a process regulated by CXCR4 and α4-integrin (41, 42). After reaching draining lymph nodes, those LCs then become immunomodulatory intermediaries that promote Treg activation and produce IL-10 (43, 44).

### Vitamin D

Vitamin D synthesis is initiated when UVB is absorbed by 7-dehydrocholesterol and converted to previtamin D3, which is then later converted to vitamin D3 (Figure 1F) (45). Most cells in the body express vitamin D receptor; hence, this molecule plays a role in numerous cellular processes including cell differentiation, cell growth inhibition, and immunomodulation (46). *In vitro* stimulation of mast cells with vitamin D suppresses IgE-mediated degranulation, while epicutaneous vitamin D administration reduces the magnitude of skin swelling in an IgE-mediated cutaneous anaphylaxis animal model (47). By promoting vitamin D3 synthesis and causing DNA damage such as CPD and 6-4PPs, UVB plays a dual role in carcinogenesis. For example, Ptch1-deficient mice are unable to produce vitamin D and demonstrate accelerated basal cell carcinoma-like tumor formation when exposed to UVR; this effect is reversed *in vivo* by exogenous supplementation of vitamin D (48). We have shown that polymorphic light eruption (PLE) patients have low levels of vitamin D in serum, however, prophylactic UVB treatment ameliorates PLE symptoms and increases vitamin D serum levels (49). A clinical trial evaluating the preventive properties of calcipotriol (a vitamin D analog) in 13 PLE patients showed that, 1 week of topical treatment with calcipotriol reduced the photoprovocative effect of simulated sun exposure and decreased severity disease score in PLE lesional skin (50).

## Langerhans cell and regulatory T-cells are the main orchestrators of UV-induced immunosuppression

LCs are a subset of dendritic cells that link the innate and adaptive immune systems by their role in priming T-cell responses upon antigen uptake. After UVR exposure, these cells migrate out of the skin and undergo changes that make them inducers of tolerance and immunosuppression (51). Compensatory mechanisms are activated after UVR exposure to repopulate the skin with LCs and rapidly recruit monocytes from blood (52). The transitory depletion of LCs is counteracted by the early recruitment of CD14^+^ monocytes (after 24 h of UVB exposure) and subsequent mobilization of two inflammatory subsets of dendritic cells (CD1a^low^CD207^−^ and CD1^low^CD207^+^ at day 1 and 4 respectively) from blood circulating cells (53). Cells of the CD11b-type Langerin^−^ phenotype are important players in the adaptive response to UVB. After irradiation, they upregulate the expression of CD86 that leads to antigen-free proliferation of Tregs and promotes the transcription of genes associated with immunotolerance (54). The skin is also populated by CD103^−^ dendritic cells that upon UVR exposure migrate into lymph nodes and induce Treg activation by the production of retinoic acid (55). Mice depleted of LCs fail to suppress CHS reactions, indicating that these cells are major players in UV induction of Tregs. This suggests that the main function of LCs is not to promote immune responses but to desensitize the skin to UV exposure (56). UVR exposure not only drives the expansion of Tregs but also restores suppressive function by inducing demethylation of the Treg genome and thereby promoting gene transcription that counteracts inflammation in skin diseases such as psoriasis (57, 58). Moreover, Treg numbers in skin increase by up to 50–60% after UV irradiation and remain in high numbers for 2 weeks after irradiation. Indeed, the impact of UV is not restricted to the skin; mice exposed to UV have CPD-positive cells in their lymph nodes for at least 4 days after exposure (59) and Tregs isolated from the blood of UV-exposed animals have a CpG hypomethylation fingerprint indicating that these Tregs were exposed to light (58). It seems that the immunosuppression triggered by UVR is in fact an adaptive response to mitigate the strong reaction of the immune system to the release of damage-associated molecular patterns (DAMPs) in favor of the repair and remodeling of damaged tissue as seen in animal models of brain injury and other trauma (60, 61). These observations suggest that the physiological immunosuppressive response to UVB that promotes the recovery of cells and damaged tissue can also be harnessed therapeutically to inflammatory diseases in body sites not exposed to UVR and therefore not only to cutaneous diseases.

## How does phototherapy work?

Photo(chemo)therapy is a first-line treatment for skin diseases of diverse etiology, including benign conditions such as psoriasis, atopic dermatitis, vitiligo, and urticaria pigmentosa (a form of mastocytosis) as well as neoplastic disorders such as mycosis fungoides. It is also used prophylactically in certain photodermatoses like PLE. Though the high efficacy of phototherapy in these diseases has long been appreciated, the exact therapeutic mechanisms have not been fully understood until now and may depend upon the type of disease for which it is prescribed. The penetration depth of UV light increases with its wavelength. Whereas most of the photons of the UVB spectrum are absorbed in the epidermis, ~30% of UVA photons do reach the upper layers of the dermis (62). The initial molecular events occurring after exposure to the different wavebands and treatments are depicted in Table 1 and include CPD formation, ROS production, UCA isomerization, vitamin D synthesis, PAF secretion, and inflammasome activation (32, 63–65, 67, 68). The phototherapeutic modalities, including UVB, UVA, and PUVA, are known for their proapototic and immunomodulatory properties, which may account for their therapeutic efficacy either alone or in combination (8).

**Table 1:** Wavebands associated with key molecular events in UV-exposed tissue.

**Molecular event**	**Causative wavebands (peak wavelength)**
CPD	UVB (300) (63)
8-MOP photoadducts	UVA (329 nm), (64)
ROS production	UVA, UVA-1, PUVA (5)
Urocanic acid isomerization	UVB (280–310 nm) (65, 66)
Vitamin D synthesis	UVB (297 nm) (67)
PAF and PAF-like molecules	UVB, UVA, PUVA (32, 68)
Inflammasome activation	UVB (1)

In particular, PUVA depletes activated CD3^+^ cells from lesional psoriatic skin by the induction of apoptosis (69, 70). The majority of CD3^+^ cells produce IL-17, a cytokine with a central role in psoriasis (71). Notably, PUVA and 311 nm UVB suppress the IL-17/IL-23 axis in both animal models and patients (72–76). Given the major role that these cells play in psoriasis pathophysiology, phototherapy's effect on them might explain (at least partially) its efficacy. But what is the fate of these activated T-cells? Are they directly eliminated by apoptosis or are they hampered by the complex immunomodulatory effects of phototherapy? Does the induction of Tregs (triggered by redundant upstream events including DNA and membrane damage as well cis-UCA formation and AhR activation) with immunosuppressive function diminish the number or the activity of those cells in skin? This is seen in psoriasis patients in whom bath PUVA therapy restores Treg functionality (77). Along this line, we have shown that CTLA-4 blockade abolishes the therapeutic effect of PUVA in a psoriasis mouse model (72). However, the systemic effect of phototherapy on the immune system and on Tregs seems therapeutically insufficient since psoriasis (78) and CTCL (79) lesions are cleared only on exposed body sites. This suggests that phototherapy must exert an additional direct local effect on keratinocytes, LCs, and/or lymphocytes among other players in the pathophysiology of those diseases, thereby allowing a local cell-to-cell interaction (between Tregs and pro-inflammatory effector T-cells) that leads to therapeutic response. For instance, on the local level, PUVA contributes to the normalization of the mTOR pathway upregulated in psoriasis (80). The systemic effect of PUVA or UVB in this disease may be completely independent of locally active mechanisms; for instance, serotonin signaling has been shown to play a crucial role in immune suppression but not inflammation or apoptosis in PUVA-exposed skin in a mouse model (81). A controversial computational model of psoriatic epidermis indicates that apoptosis of stem and transit amplifying cells after exposure to 311 nm UVB alone may be sufficient to clear lesional skin, suggesting that direct keratinocyte apoptosis is a key therapeutic mechanism (82). Moreover, psoriatic lesions clinically cleared after phototherapy contain residual oligoclonal T-cell populations that share features of T_RM_ and are capable of producing IL-17. These cells are likely responsible for the initiation of recurrent flares in the same body locations, implying that clinical resolution after phototherapy does not depend on depletion of cells with a dysregulated phenotype (83). In any case, the difficulty inherent in evaluating the roles of direct apoptosis and immunosuppression independently of each other highlights the need to investigate and compare phototherapy against other therapeutic approaches that induce one effect or the other.

In atopic dermatitis, phototherapy may work by strengthening the skin barrier function of lesional skin, shifting the expression of epidermal proteins like filaggrin, loricrin, and involucrin (84), augmenting levels of AMPs, (85) and shifting the microbiome diversity, among other effects (86, 87). In vitiligo, 311 UVB and PUVA directly stimulate the proliferation of melanocytes and by inducing Tregs help overcome the autoimmune pathophysiology of this disease by controlling cellular mediated cytotoxicity against pigment-producing cells (88). In mastocytosis, phototherapy might act by direct cytotoxicity against activated mast cells and by stabilizing mast cells, thus inhibiting them from releasing soluble proinflammatory mediators such as histamine (89). In graft vs. host disease (GVHD), the predominant mechanism of action may be immunomodulation by downregulating the activity of grafted cells against the host (90). The effect of OVB in pruritus remains entirely elusive at the moment; however, a halfside comparison study implied a systemic effect, since treatment reduced pruritus not only on the irradiated body half but also to an equal degree on the unirradiated side (91). UVB-induced reduction of systemic levels of pro-pruritic IL-31 may be involved (92).

In the prophylaxis of photodermatoses such as PLE, phototherapy may act by inducing melanization in the skin, increasing vitamin D levels, restoring the susceptibility of the skin to respond to UV by depleting LCs and allowing infiltration of neutrophils, restoring the abnormal chemotactic potential of neutrophils, and increasing the number of peripheral Tregs to overcome the impaired immunosuppressive function of these cells (93, 94). Moreover, recent work has indicated that mast cells play a crucial role in countering itch by inducing phototolerance after photohardening treatment with increased numbers of Tregs in blood (95–97).

The efficacy of phototherapy in the most common form of CTCL, mycosis fungoides (MF), depends on the severity of the disease and on the type of presenting lesions. UVB has a high success rate in patients with patch-stage lesions, whereas PUVA is also effective in patients with plaque- and even early tumor-stage lesions. This differential response may be attributed to the lower penetration capacity of UVB compared to UVA as used in PUVA photochemotherapy. Alternatively, PUVA may induce longer lasting photoproducts than UVB does, resulting in a sustained downstream immunosuppressive cascade. Notably, phototherapy with both PUVA or UVB is effective not only in MF but also in lymphomatoid papulosis (LyP) (98), a disease that sometimes coexists with MF and is characterized by papules and nodules with deep skin infiltration up to 1 cm or more; however, these light treatments only directly reach the infiltrating cells in the most superficial layers but not those in the diseased deep tissue. The immunosuppressive microenvironment induced by phototherapy in the upper layers of the skin may be sufficient to deplete infiltrating cells in LyP and/or prevent the occurrence of new lesions in this intermittent disease.

Although broad band UVB, narrow band UVB, and oral or topical PUVA lead to different photoproducts at the DNA level (CPD vs. psoralen-DNA photoadducts) and produce overlapping molecular events (such as PAF and PAF like molecules) (Table 1), they have similar downstream effects including the induction of apoptosis and the downregulation of immune responses (including the induction of Tregs locally and systemically). In contrast, exposure to UVA and UVA1 (340–400 nm) mainly leads to oxidative alterations at the DNA and membrane level and elicits cellular responses such as the induction of MMPs and collagenase, mediators that are important particularly in UVA1's therapeutic action in fibrotic skin conditions including morphea and sclerodermic chronic GVHD (99). This may be due to the downregulation of TGFβ signaling transducers in the skin after UVA1 exposure (100).

## Concluding remarks

After more than 100 years of using simple artificial UV light therapeutically, beginning with the pioneering work of Nobel laureate Finsen in treating cutaneous tuberculosis, diverse photo(chemo)therapeutic modalities have evolved to treat a wide spectrum of skin diseases and to prevent photodermatoses. During this evolutionary process, photo(chemo)therapy has offered avenues to better understand disease and therapeutic mechanisms and provided a large body of evidence for refining therapeutic strategies in the future. In this context, our research with PUVA has led us to realize the potential role of IL-9 in psoriasis and CTCL. PUVA reduces levels of IL-9 and IL-17 in both the TGFβ transgenic and imiquimod psoriasis mouse model (72, 75). The blockade of IL-9 (101) or IL-17 (72) reduced the psoriatic phenotype of these mice. Meanwhile, IL-17 antibody blockers have reached the market and are currently considered the most powerful anti-psoriatic treatment. And now, in light of evidence that PUVA also downregulates IL-9 in CTCL patients and that anti-IL9 treatment reduces tumor growth in a CTCL mouse model (102), IL-9 targeting has become a promising therapeutic intervention in patients with CTCL. These and other advances in the understanding of phototherapeutic mechanisms in inflammatory and neoplastic diseases will help to make therapeutic strategies more precise, targeted, personalized, safe, and efficient.

## Author contributions

PV-G and PW conceived the ideas and drafted the manuscript. PV-G drafted the figure. Both authors revised and approved the final version of the manuscript for publication.

### Conflict of interest statement

The authors declare that the research was conducted in the absence of any commercial or financial relationships that could be construed as a potential conflict of interest.
